# Sweroside Alleviated LPS-Induced Inflammation via SIRT1 Mediating NF-κB and FOXO1 Signaling Pathways in RAW264.7 Cells

**DOI:** 10.3390/molecules24050872

**Published:** 2019-03-01

**Authors:** Rui Wang, Zhaoyue Dong, Xiaozhong Lan, Zhihua Liao, Min Chen

**Affiliations:** 1 College of Pharmaceutical Sciences, Key Laboratory of Luminescent and Real-Time Analytical Chemistry (Southwest University), Ministry of Education, Southwest University, Chongqing 400715, China; liuchishou@email.swu.edu.cn (R.W.); dzy2018@email.swu.edu.cn (Z.D.); 2 TAAHC-SWU Medicinal Plant R&D Center, XiZang Agriculture and Animal Husbandry College, Nyingchi, Tibet 860000, China; lanxiaozhong@163.com; 3 School of Life Sciences, Southwest University, Chongqing 400715, China; zhliao@swu.edu.cn

**Keywords:** sweroside, *Pterocephalus hookeri*, SIRT1, NF-κB, FOXO1, inflammation

## Abstract

*Pterocephalus hookeri* was used as a traditional Chinese medicine for the treatment of rheumatoid arthritis. Sweroside was a main iridoid isolated from *P. hookeri*. The present study aimed to investigate the anti-inflammatory effect mechanism of sweroside. In RAW264.7 cells induced by lipopolysaccharide (LPS), the abnormal proliferation, the NO content increase, and the downregulated Sirtuin1 (SIRT1) expression were observed. Sweroside could alleviate the inflammation by inhibiting cell proliferation through arresting the cell cycle at the G0/G1 phase, by suppressing pro-inflammatory cytokines and by promoting anti-inflammatory cytokines in LPS-induced RAW264.7 cells. Further mechanism research indicated that sweroside could activate the SIRT1, then suppress the nuclear factor-kappa B (NF-κB) and promote the Forkhead transcription factor O1 (FOXO1) signaling pathways. The present study indicated that sweroside may be the main anti-inflammatory constituent of *P. hookeri* and a promising candidate for anti-inflammation therapy.

## 1. Introduction

Rheumatoid arthritis (RA) is a chronic autoimmune disease which expresses as a symmetric polyarthritis associated with swelling and pain in multiple joints [1]. RA significantly hampered the quality of people’s lives, covering 1% of the world’s population and leading to severe disability in the patients [2]. It was believed that inflammation have been involved in the development and progression of RA [3]. Therefore, the inhibition of inflammation was an effective way to treat RA. Inflammation was a protective response of the immune system which would resist exogenous infection, recur the injury tissue and eliminate invading pathogens. However, disordered inflammation might result in secondary damage and immune pathology, deteriorating diseases such as RA [4]. Therefore, the development of effective anti-inflammatory drugs occupied the core position for the treatment of RA.

*Pterocephalus hookeri*, a traditional medicine recorded in Chinese Pharmacopoeia (2015 version), had been widely used for the treatment of RA [5]. Currently, it was considered that iridoids were the main anti-inflammatory constituents in this herb [6]. Sweroside, isolated and identified from *P. hookeri* [7], was a typical iridoid that exhibits diverse biological activities, such as hepatoprotective [8], antidiabetic [9] and anti-inflammatory [10] effects.

Sirtuin1 (SIRT1) was a member of the sirtuin family, and it was an enzyme responsible for the deacetylation of proteins to extend the life span and promote the health of a wide variety of organisms from yeast to mammals [11]. Nearly, it was reported that SIRT1 was closely related to inflammation [12]. Herein, we aimed to investigate the anti-inflammatory effect of sweroside and its mechanisms regulated by SIRT1.

## 2. Results

### 2.1. The Effects of NO Production and Morphology in LPS-Induced RAW264.7 Cells

**Figure 1:**
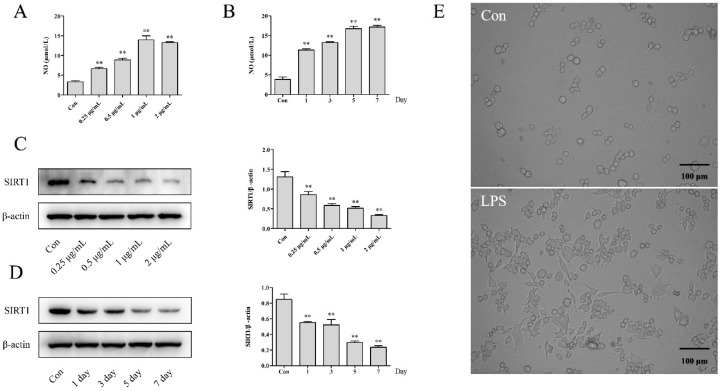
The effects of lipopolysaccharides (LPS) on the NO, SIRT1 and morphology observation in RAW264.7 cells: (**A**) The NO assay was measured with LPS (0.25–2 µg/mL) treatment for 24 h. (**B**) The NO assay was measured with 1 µg/mL LPS treatment for 1, 3, 5 and 7 days. (**C**) The expression of SIRT1 was detected with LPS (0.25–2 µg/mL) treatment for 24 h. (**D**) The expression of SIRT1 was detected with 1 µg/mL LPS treatment for 1, 3, 5 and 7 days. (**E**) The morphological changes of RAW264.7 cells treated with 1 µg/mL LPS for 5 days were observed using a microscope at 200×. The results were expressed as mean ± SD (*n* = 3), * *p* < 0.05, ** *p* < 0.01 compared with the Con group.

The NO assay showed that lipopolysaccharide (LPS) could significantly increase the production of NO with dose-dependent (0.25, 0.5, 1 and 2 μg/mL) and time-dependent manners (1 μg/mL LPS for 1, 3, 5 and 7 days), compared with the Con group (Figure 1A,B, *p* < 0.01). Meanwhile, the morphological observation in the LPS group (1 μg/mL, 5 days) exhibited obvious proliferation with an increasing size and irregular pseudopods, indicating the inflammation leading the abnormal proliferation in RAW264.7 cells, as shown in Figure 1E.

### 2.2. The Effects on the Expression of SIRT1 in LPS-Induced RAW264.7 Cells

As presented in Figure 1C,D, the expression of SIRT1 was downregulated significantly with exposure to LPS, both in dose-dependent and time-dependent manners (*p* < 0.01). The results declared that the inflammation induced by LPS would inhibit the expression of SIRT1.

### 2.3. The Effects of Cell Proliferation and NO Production on Sweroside Treatment in LPS-Induced RAW264.7 Cells

**Figure 2:**
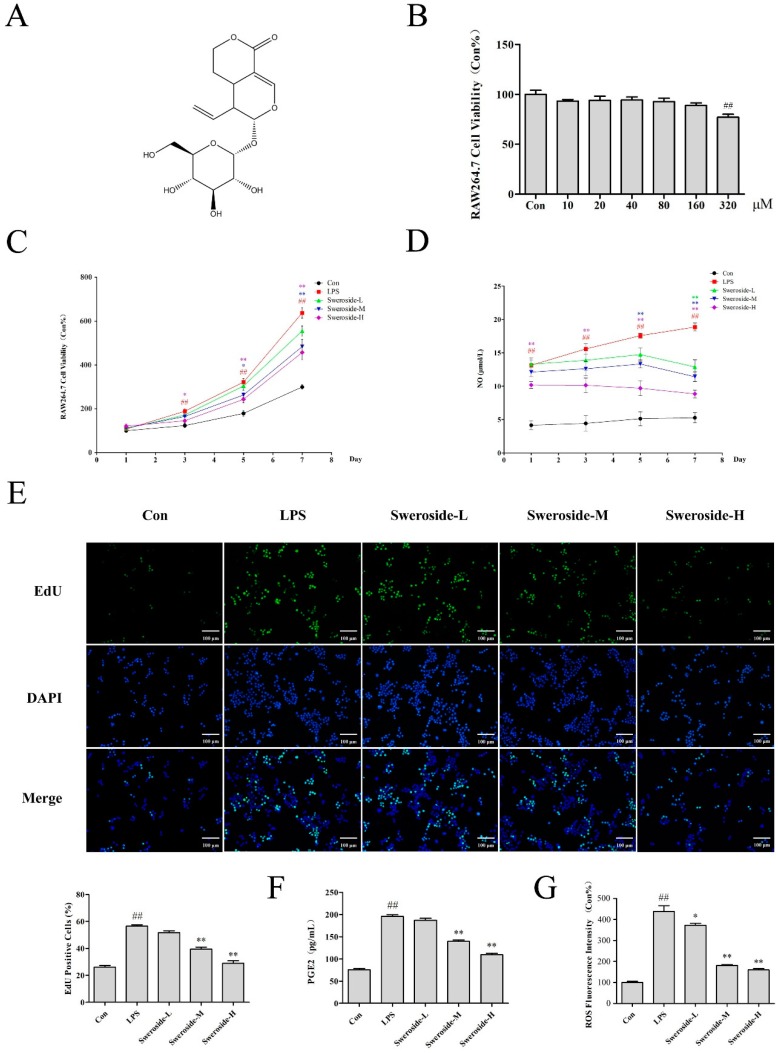
The effects of sweroside in LPS-induced RAW264.7 cells: (**A**) The chemical structure of sweroside. (**B**) The cytotoxicity assay was conducted to measure the viability of RAW264.7 cells with sweroside (10–320 µM) for 24 h. (**C**) The proliferation assay was conducted to measure the viability of sweroside (20, 40 and 80 μM) in LPS-induced RAW264.7 cells for 7 days. (**D**) The NO assay was detected with the treatment of sweroside (20, 40 and 80 μM) in LPS-induced RAW264.7 cells for 7 days. (**E**) The EdU assay was detected with the treatment of sweroside (20, 40 and 80 μM) in LPS-induced RAW264.7 cells for 5 days via a fluorescence microscope at 200×. The prostaglandin E2 (PGE2) (**F**) and reactive oxygen species (ROS) (**G**) assay was detected with the treatment of sweroside (20, 40 and 80 μM) in LPS-induced RAW264.7 cells for 5 days. The results were expressed as mean ± SD (*n* = 3), ^#^ *p* < 0.05, ^##^ *p* < 0.01 compared with the Con group; * *p* < 0.05, ** *p* < 0.01 compared with the LPS group.

The extract of *Pterocephalus hookeri* for 95% ethanol exhibited weak cytotoxicity and an obvious anti-inflammatory activity in the pre-experiment (S1. Experiments and S2. Results of the Supplementary Materials). Therefore, the subsequent researches were mainly focused on sweroside, which was the main constituent of *P. hookeri*. The cytotoxicity assay indicated that sweroside exhibited no significant cytotoxicity within the concentrations 0–160 μM, while there was a weak cytotoxicity (cell viability at 77.0%) at 320 μM (Figure 2B). In further anti-inflammation research, 20, 40 and 80 μM were set as the low, middle and high dosages, respectively. Our results showed that sweroside could significantly reverse the abnormal proliferation with a dose-independent manner in LPS-induced RAW264.7 from the 3rd to 7th days (*p* < 0.01, Figure 2C). The 5-ethynyl-2’-deoxyuridine (EdU) assay for 5 days also revealed a significant anti-proliferative effect of sweroside, with an obvious decrease in green fluorescent cells marked by EdU (Figure 2E). Meanwhile, NO, as an important pro-inflammatory molecule, was decreased from the 1st to 7th days in sweroside-treated groups, especially in the high-dose group (*p* < 0.01, Figure 2D).

### 2.4. The Effects of PGE2 and ROS on Sweroside Treatment in LPS-Induced RAW264.7 Cells

The inflammatory indicators prostaglandin E2 (PGE2) and reactive oxygen species (ROS) in RAW264.7 cells were detected on the 5th day. The result showed that the levels of PGE2 and ROS in the LPS group increased significantly compared with Con group (*p* < 0.01), while the treatment of sweroside significantly decreased the levels of PGE2 and ROS with a dose-dependent manner (*p* < 0.05 or 0.01, Figure 2F,G).

### 2.5. The Effects of Apoptosis and Cell-Cycle Distribution on Sweroside Treatment in LPS-Induced RAW264.7 Cells

**Figure 3:**
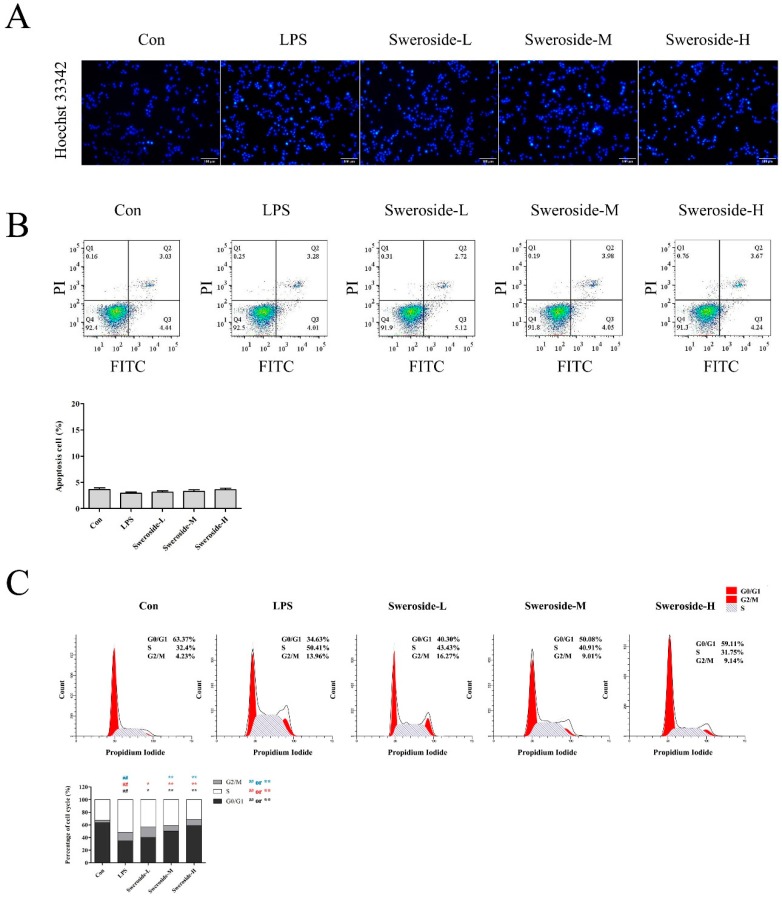
The apoptosis and cell-cycle distribution effects of sweroside in LPS-induced RAW264.7 cells: (**A**) The Hoechst 33342 stain for apoptosis was detected with the treatment of sweroside (20, 40 and 80 μM) in LPS-induced RAW264.7 cells for 5 days via a fluorescence microscope at ×200. (**B**) The flow cytometer analysis for apoptosis was detected with the treatment of sweroside (20, 40 and 80 μM) in LPS-induced RAW264.7 cells for 5 days. (**C**) The flow cytometer analysis for cell-cycle distribution was detected with the treatment of sweroside (20, 40 and 80 μM) in LPS-induced RAW264.7 cells for 5 days. The results were expressed as mean ± SD (*n* = 3), ^#^ *p* < 0.05, ^##^ *p* < 0.01 compared with the Con group; * *p* < 0.05, ** *p* < 0.01 compared with the LPS group.

As shown in Figure 3A,B, no obvious apoptosis was observed in the LPS- or sweroside-treatment groups, both in the Hoechst 33342 stain and in the flow cytometer analysis (*p* > 0.05).

To further investigate the effect of sweroside on proliferative inhibition, the cell-cycle distribution was evaluated (Figure 3C). The flow cytometer assay revealed that the percentage of the G0/G1 phase were increased significantly after the treatment of sweroside in LPS-induced RAW264.7 cells with a dose-dependent manner (*p* < 0.01).

### 2.6. The Effects of SIRT1/NF-κB on Sweroside Treatment in LPS-Induced RAW264.7 Cells

**Figure 4:**
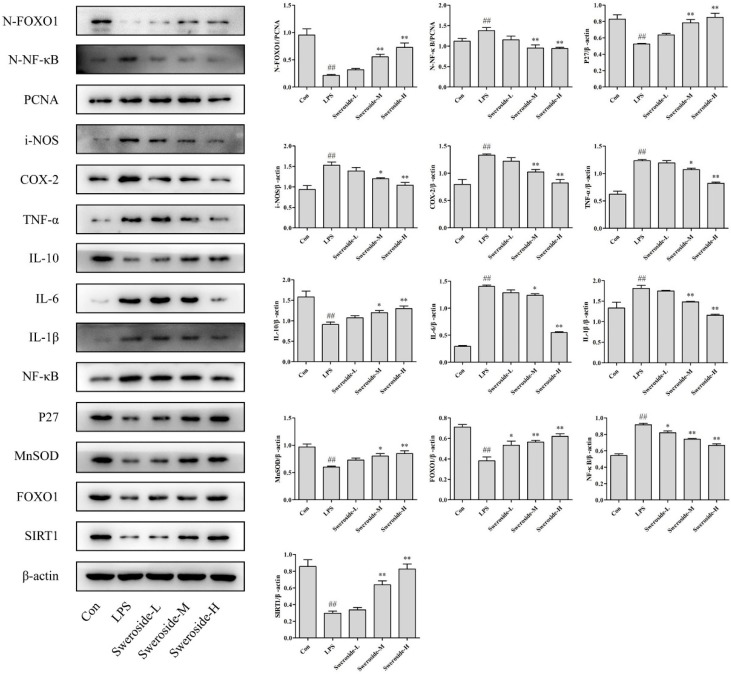
The Western blot analysis for sweroside in LPS-induced RAW264.7 cells: SIRT1, NF-κB, FOXO1, N-NF-κB, N-FOXO1, i-NOS, COX-2, IL-1β, IL-6, IL-10, TNF-α, MnSOD and P27 were detected with the treatment of sweroside (20, 40 and 80 μM) in LPS-induced RAW264.7 cells for 5 days. ^#^ *p* < 0.05, ^##^ *p* < 0.01 compared with the Con group; * *p* < 0.05, ** *p* < 0.01 compared with the LPS group.

As shown in Figure 4, the expression of SIRT1 was upregulated with the sweroside administration in LPS-induced RAW264.7 cells. In addition, the Nuclear factor-kappa B (NF-κB) and NF-κB in nucleus (N-NF-κB) were also downregulated significantly after the treatment of sweroside. As downstream signals of NF-κB, the level of inflammatory mediators inducible-NOS (i-NOS); cyclooxygenase-2 (COX-2); and pro-inflammatory cytokines tumor necrosis factor-α (TNF-α), interleukin-1β (IL-1β) and interleukin-6 (IL-6) were decreased obviously after the treatment of sweroside. Only the level of anti-inflammatory cytokine interleukin-10 (IL-10) was strengthened with the treatment of sweroside (*p* < 0.05 or 0.01).

### 2.7. The Effects of SIRT1/FOXO1 on Sweroside Treatment in LPS-Induced RAW264.7 Cells

Forkhead transcription factor O1 (FOXO1) was upregulated significantly with the sweroside treatment in LPS-induced RAW264.7 cells. Meanwhile, the FOXO1 in nucleus (N-FOXO1) was also upregulated obviously. As downstream signals of FOXO1, the expressions of P27 and MnSOD were also increased after the treatment of sweroside (Figure 4, *p* < 0.05 or 0.01).

### 2.8. The Effects of NAM on Sweroside Treatment in LPS-Induced RAW264.7 Cells

**Figure 5:**
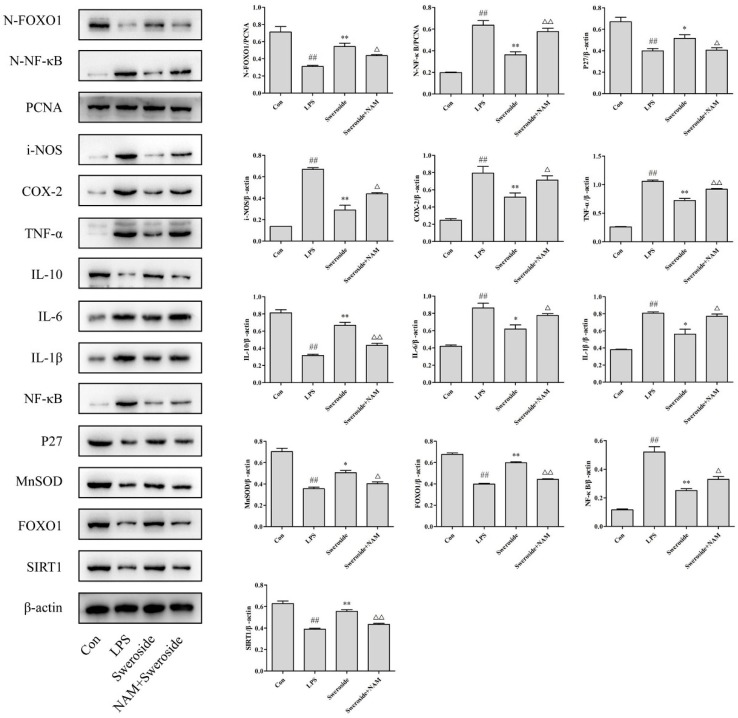
The Western blot analysis for sweroside and nicotinamide (NAM) in LPS-induced RAW264.7 cells: SIRT1, NF-κB, FOXO1, N-NF-κB, N-FOXO1, i-NOS, COX-2, IL-1β, IL-6, IL-10, TNF-α, MnSOD and P27 were detected with the treatment of sweroside (80 μM) and NAM (20 μM) in LPS-induced RAW264.7 cells for 5 days. ^#^ *p* < 0.05, ^##^ *p* < 0.01 compared with the Con group; * *p* < 0.05, ** *p* < 0.01 compared with the LPS group; ^△^ *p* < 0.05, ^△△^ *p* < 0.01 compared with the sweroside group.

As an inhibitor of SIRT1, 20 μM of nicotinamide (NAM) was applied to further investigate the anti-inflammatory mechanism of sweroside (Figure 5). The results showed that the administration of NAM reversed the expressions of all proteins significantly, including SIRT1, NF-κB, N-NF-κB, FOXO1, N-FOXO1, i-NOS, COX-2, IL-1β, IL-6, IL-10, TNF-α, MnSOD and P27, compared with the sweroside-treated groups (*p* < 0.05 or 0.01).

## 3. Discussion

As a sufficiently speedy response of the immune system, inflammation could be found with the progression of many diseases with symptoms of redness, swelling, fever and pain [13]. The immune system, which consists of innate immunity and specific immunity, acts as a soldier to protect the organisms against multiple diseases. Macrophages are intrinsic innate immune cells, which uses phagocytosis to play an important role in immunity and inflammation [14]. Appropriate phagocytosis in macrophages could resist foreign invasion and maintain homeostasis. However, an excess of macrophages would greatly enhance phagocytosis and continuously release inflammatory cytokines, which would aggravate the damage surrounding tissue [15]. Rheumatoid arthritis (RA), a typical autoimmune disease, is caused by the excessive immune system and sustained inflammation [4]. Therefore, the regulation of macrophages in homeostasis is an effective way to alleviate abnormal immune responses with persistent inflammation and tissue damage [16].

LPS is a complex glycolipid in the outer membrane of Gram-negative bacteria, which are utilized to induce inflammatory models [17]. Recently, SIRT1, which was an NAD^+^-dependent class III protein deacetylase, was found to regulate inflammation through the deacetylation of critical transcription factors [18]. Our results indicated that LPS decreased the expression of SIRT1 in RAW264.7 cells, which was consistent with the previous report [19], suggesting the activation of SIRT1 might be a promising therapeutic strategy for inflammation.

As an inflammatory indicator, NO was synthesized by nitric oxide synthases (NOSs), and only i-NOS was activated in response to inflammatory stimuli [20]. PGE2, generated by the metabolism of arachidonic acid through the COX, was another major indicator in inflammation. COX-1 and COX-2 were the two isoforms of COX. Only COX-2 was an inducible isoform of COX that participated in inflammation [21]. Besides, inflammation cytokines TNF-α, IL-6, IL-1β and IL-10 [22] are also involved in regulating the inflammatory and immune response. The present study indicated that sweroside could alleviate inflammation by reversing these inflammatory mediators and cytokines effectively in LPS-induced RAW264.7 cells.

**Figure 6:**
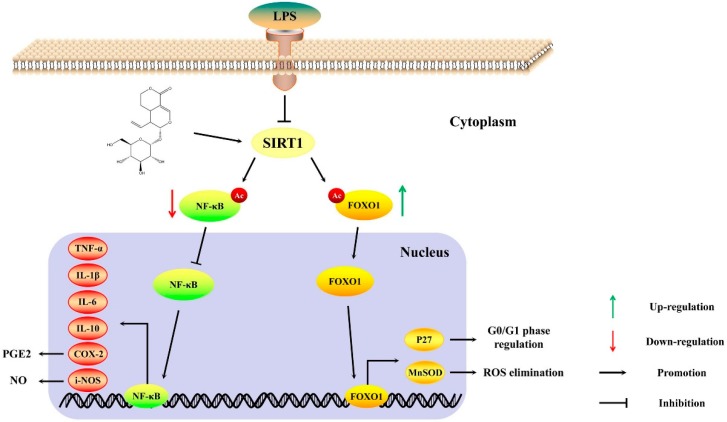
The possible anti-inflammation mechanism of sweroside in LPS induced-RAW264.7 cells.

To further investigate the molecular mechanism of sweroside, we focused on SIRT1 and its downstream signaling pathways. It was commonly recognized that SIRT1 exerted anti-inflammatory effects by the deacetylation of transcriptional regulators, particularly NF-κB and FOXO1 [11]. Upon certain stimuli, such as LPS, NF-κB was phosphorylated and transported into the nucleus to regulate a variety of inflammatory genes expression, containing i-NOS, COX-2, IL-1β, TNF-α, IL-10 and IL-6 [23]. The deacetylation of NF-κB regulated by SIRT1 would inhibit the expression of its downstream signaling pathways and then alleviate inflammation [24]. Our results proved that sweroside increased the expression of SIRT1, inhibiting the expression and transcription of NF-κB and reversing the inflammatory mediators and cytokines. After the administration of NAM, the inhibitor of SIRT1, the expression of NF-κB-regulated pathways were reversed partly, which further confirmed that the ant-inflammatory effect of sweroside through the NF-κB singling pathway could be mediated by SIRT1 (Figure 6).

The excessive phagocytosis for macrophages could aggravate inflammation and tissue damage [15]. Therefore, the proper regulation of macrophages is an effective means to relieve inflammation. In present study, sweroside significantly decreased the cell viability in LPS-induced RAW264.7 cells via arresting the cell cycle in the G0/G1 phase. FOXO1 was one of the isoforms in the FOXO family and played a critical role in the cell proliferation and differentiation [25]. The activation of SIRT1 could promote the expression of FOXO1 and the transcription of FOXO1 from the cytoplasm into the nucleus [26]. Acting as a transcription factor, FOXO1 regulated the process of the G0/G1 phase in the cell cycle by controlling the transcription of P27 (a cyclin-dependent kinase inhibitor) [27]. Meanwhile, as a key ROS scavenger, MnSOD was upregulated by FOXO1 to relieve oxidative stress [28], which was also beneficial in alleviating inflammation. Our results proved that sweroside increased the expression of SIRT1, promoting the expression and transcription of FOXO1, upregulating the expression of P27 and MnSOD and decreasing the cell viability in LPS-induced RAW264.7 cells. After the administration of NAM, the expression of FOXO1-regulated pathways were also reversed partly, explicating that the proliferative inhibition of sweroside through FOXO1 signaling pathways could be mediated by SIRT1 (Figure 6).

## 4. Materials and Methods

### 4.1. Chemicals and Regents

Sweroside (purity > 98%, Figure 2A) was obtained from National Institutes for Food and Drug Control (Beijing, China). Fetal bovine serum (FBS) and phosphate buffer saline (PBS) were acquired from Servicebio Company (Wuhan, China). DMEM, DMSO, trypsin, the RIPA Lysis buffer and the PVDF membrane were obtained from Solarbio Company (Beijing, China). The annexin V-FITC apoptosis detection kit was obtained from Vazyme Biotech Company (Nanjing, China). Hoechst 33342, NAM, SIRT1, NF-κB, β-actin and the proliferating cell nuclear antigen (PCNA) antibodies were from Beyotime Institute of Biotechnology (Shanghai, China). The TNF-α, IL-1β, IL-6, IL-10, MnSOD, FOXO1, COX-2 and i-NOS antibodies were obtained from Proteintech Company (Wuhan, China). The ROS assay kit, CCK-8 assay kit, NO assay kit, cell cycle and apoptosis analysis kits were from Beyotime Institute of Biotechnology (Shanghai, China). The PGE2 Elisa Kit was purchased from Hui Jia Company (Xiamen, China). The other chemicals were of analytical grade and were commercially available.

### 4.2. Cell Culture and Treatment

RAW264.7 cells were obtained from the Shanghai Institute of Cell Biology, Chinese Academy of Sciences (Shanghai, China) and cultured in DMEM supplemented with 10% FBS, 100 U/mL penicillin and streptomycin. The cells were incubated in a humidified chamber containing 5% CO_2_ at 37 °C. DMEM was replenished every 2 or 3 days.

### 4.3. Cytotoxicity Assay

Cell cytotoxicity assay was performed using the CCK-8 method. The RAW264.7 cells were seeded in a 96-well plates at a density of 5000 per well with 100 μL DMEM for 12 h to adhere. The normal control cells were set in the Con group treated with DMSO, whereas cells in the experimental groups were exposed to 10, 20, 40, 80, 160 and 320 μM sweroside for 24 h. For all groups, the final concentration of DMSO added to the cells was <0.1%. Each well was added 10 μL CCK-8 for 4 h and then read by the multifunctional microplate reader (Gene Company, Hong Kong, China). All the procedures were according to the manufacturer’s instructions.

### 4.4. Determination of NO Production

The RAW264.7 cells were seeded in 48-well plates at a density of about 8000 per well with 200 μL complete DMEM for 12 h until adherence. The normal control cells were set in the Con group. The level of NO with different treatments in each group were determined by the NO assay kit (Beyotime Institute of Biotechnology, Shanghai, China) with the multifunctional microplate reader. All the procedures were according to the manufacturer’s instructions.

### 4.5. Microscopic Observation

The morphological observation associated with LPS-induced RAW264.7 cells (1 μg/mL) for 5 days were assessed using a fluorescence microscope (Olympus, Tokyo, Japan). The images were captured at a magnification of 200×.

### 4.6. Cell Proliferation Assay for Treatment of Sweroside in LPS-Induced RAW264.7 Cells

The cell proliferation assay was assessed using the CCK-8 and EdU methods. The RAW264.7 cells were seeded in 48-well plates at a density of about 8000 per well. After a 12 h adherence, low (L), middle (M) and high (H) doses of sweroside (20, 40 and 80 μM) were added to the LPS (1 μg/mL)-induced RAW264.7 cells for 7 days and the cell viability was detected for every two days. The normal control cells were set in the Con group. Furthermore, individual proliferating cells could be intuitively observed by EdU analysis. The RAW264.7 cells were seeded in 48-well plates with the same treatment of the CCK-8 assay. After 5 days, the EdU positive cells were visualized using the fluorescence microscope. All the procedures were according to the manufacturer’s instructions.

### 4.7. PGE2 and ROS Analysis

The RAW264.7 cells were seeded into 6-well plates with a density of 1 × 10^6^ per well. After 12 h of adherence and treated the same as the proliferation analysis for 5 days, The PGE2 and ROS in each group were determined by the PGE2 ELISA kit (Hui Jia Company, Xiamen, China) and the ROS assay kit (Beyotime Institute of Biotechnology, Shanghai, China) according to the manufacturer’s instructions.

### 4.8. Cell Apoptotic Analysis by Hoechst 33342 Stain and Flow Cytometer

The RAW264.7 cells were seeded in 6-well plates with the same treatment as in the PGE2 and ROS analyses. After 5 days, all groups were washed with PBS two times and incubated with the Hoechst 33342 stain at 4 °C for 20 min. After staining, the nuclear morphological changes were observed through the fluorescence microscope. Another cell apoptotic analysis was detected by flow cytometer. After the treatment of sweroside, all groups were washed with PBS and digested with trypsin. After collection by centrifugation at 1500 rpm/min, each sample was suspended in 100 μL buffer, mixed with 5 μL propidium iodide (PI) and 5 μL FITC and kept in the dark at room temperature for 20 min. Then, the samples were added another 400 μL buffer and analyzed using flow cytometer (BD FACSVerse, Shanghai, China).

### 4.9. Cell-Cycle Distribution Analysis by Flow Cytometer

The RAW264.7 cells were treated the same as in the apoptotic analysis. After digestion and collection, the cells were washed with cold PBS and fixed with 70% ice-cold ethanol storing at 4 °C overnight. Prior to analysis, the cells were washed twice with cold PBS and incubated with RNase for 30 min to eliminate the RNA. Lastly, all samples were stained with PI for 30 min and analyzed on a flow cytometer.

### 4.10. Western Blot Analysis

The RAW264.7 cells with different treatments were lysed in a RIPA lysis buffer to get the proteins. The protein concentration was determined using a BCA protein assay kit. Then, equal amounts of protein were suspended in a SDS-PAGE loading buffer and heated at 100 °C for 5 min. After that, all sample proteins were separated by electrophoresis on 10% or 12% SDS-PAGE. Then, the proteins were transferred into PVDF membranes. The membranes were blocked for 2 h with 5% skimmed milk at room temperature. After blocking, the membranes were incubated with the corresponding antibodies (SIRT1, NF-κB, FOXO1, i-NOS, COX-2, IL-1β, IL-6, IL-10, TNF-α, PCNA and β-actin) at a dilution of 1:1000 overnight at 4 °C. Then, peroxidase-conjugated secondary antibodies were incubated with the membranes for 1 h. The results were observed via an imaging system (Tanon 5200, Shanghai, China).

### 4.11. Statistical Analysis

All data were shown as mean ± SD with the analysis by the GraphPad Prism version 5.0 software (GraphPad Software, San Diego, CA, USA). The significant differences within the groups were analyzed by a one-way analysis of variance (ANOVA). The comparison between two groups was judged by the *t*-test. The significance was defined as *p* < 0.05 (*, ^#^ or ^△^) and *p* < 0.01(**, ^##^ or ^△△^).

## 5. Conclusions

The present study revealed that sweroside could ameliorate LPS-induced inflammation in RAW264.7 cells through the activation of SIRT1 to regulate SIRT1/NF-κB and SIRT1/FOXO1 signaling pathways. Based on these studies, it is possible to provide a basic research for other cell lines and in vivo model of rheumatoid arthritis with further investigation, supporting sweroside as a potential anti-inflammatory medicine in pharmacologic theory. It also provided the evidence that sweroside may be a main anti-inflammatory constituent of *P. hookeri*.

## Supplementary Materials

The followings are available online, S1. Experiments 1.1 Cytotoxic activity test of *P. hookeri* extract in RAW264.7 cells. 1.2 Anti-inflammatory test of *P. hookeri* extract in LPS-induced RAW264.7 cells. S2. Results 2.1 Effects of cytotoxicity and anti-inflammatory activity on *P. hookeri* extract. Figure S2 Effects of cytotoxic activity and anti-inflammation on *P. hookeri* extract in RAW264.7 cells.

## References

[1] Zhang T., Li H., Shi J., Li S., Li M., Zhang L., Zheng L., Zheng D., Tang F., Zhang X. (2016). p53 predominantly regulates IL-6 production and suppresses synovial inflammation in fibroblast-like synoviocytes and adjuvant-induced arthritis. Arthritis. Res. Ther..

[2] Firestein G.S. (2003). Evolving concepts of rheumatoid arthritis. Nature.

[3] Mateen S., Zafar A., Moin S., Khan A.Q., Zubair S. (2016). Understanding the role of cytokines in the pathogenesis of rheumatoid arthritis. Clin. Chim. Acta.

[4] Weyand C.M., Zeisbrich M., Goronzy J.J. (2017). Metabolic signatures of T-cells and macrophages in rheumatoid arthritis. Curr. Opin. Immunol..

[5] Zhang L., Hu J., Lin J., Fang W., Du G. (2009). Anti-inflammatory and analgesic effects of ethanol and aqueous extracts of *Pterocephalus hookeri* (C.B. Clarke) Höeck. J. Ethnopharmacol..

[6] Chen Y., Yu H., Guo F., Wu Y., Li Y. (2018). Antinociceptive and anti-inflammatory activities of a standardizedextract of bis-iridoids from *Pterocephalus hookeri*. J. Ethnopharmacol..

[7] Tang C., Fan G., Li Q., Su J., Meng X., Zhang Y. (2017). Simultaneous determination of ten compounds in two main medicinal plant parts of Tibetan herb, *Pterocephalus hookeri* (CB Clarke) Höeck, by ultra-high performance liquid chromatography-photodiode array. Trop. J. Pharm. Res..

[8] Yang Q.L., Yang F., Gong J.T., Tang X.W., Wang G.Y., Wang Z.T., Yang L. (2016). Sweroside ameliorates α-naphthylisothiocyanate-induced cholestatic liver injury in mice by regulating bile acids and suppressing pro-inflammatory responses. Acta Pharmacol. Sin..

[9] Mahendran G., Thamotharan G., Sengottuvelu S., Narmatha Bai V. (2014). Anti-diabetic activity of *Swertia corymbosa* (Griseb.) Wight ex C.B. Clarke aerial parts extract in streptozotocin induced diabetic rats. J. Ethnopharmacol..

[10] Zhang R., Wang C., Jiang H., Tian X., Li W., Liang W., Yang J., Zhong C., Chen Y., Li T. (2018). Protective Effects of Sweroside on IL-1β-Induced Inflammation in Rat Articular Chondrocytes Through Suppression of NF-κB and mTORC1 Signaling Pathway. Inflammation.

[11] Haigis M.C., Sinclair D.A. (2010). Mammalian sirtuins: Biological insights and disease relevance. Annu. Rev. Pathol..

[12] Rajendrasozhan S., Yang S., Kinnula V.L., Rahman I. (2008). SIRT1, an Antiinflammatory and Antiaging Protein, Is Decreased in Lungs of Patients with Chronic Obstructive Pulmonary Disease. Am. J. Resp. Crit. Care.

[13] Ferrero-Miliani L., Nielsen O.H., Andersen P.S., Girardin S.E. (2006). Chronic inflammation: Importance of NOD2 and NALP3 in interleukin-1β generation. Clin. Exp. Immunol..

[14] Underhill D.M., Goodridge H.S. (2012). Information processing during phagocytosis. Nat. Rev. Immunol..

[15] Papathanassiu A.E., Ko J., Imprialou M., Bagnati M., Srivastava P.K., Vu H.A., Cucchi D., McAdoo S.P., Ananieva E.A., Mauro C. (2017). BCAT1 controls metabolic reprogramming in activated human macrophages and is associated with inflammatory diseases. Nat. Commun..

[16] Rajendran P., Chen Y., Chen Y., Chung L., Tamilselvi S., Shen C., Day C.H., Chen R., Viswanadha V.P., Kuo W. (2018). The multifaceted link between inflammation and human diseases. J. Cell. Physiol..

[17] Bascands J., Bachvarova M., Neau E., Schanstra J.P., Bachvarov D. (2009). Molecular determinants of LPS-induced acute renal inflammation: Implication of the kinin B_1_ receptor. Biochem. Biophys. Res. Commun..

[18] Xie J., Zhang X., Zhang L. (2013). Negative regulation of inflammation by SIRT1. Pharmacol. Res..

[19] Zhang H., Shan Y., Wu Y., Xu C., Yu X., Zhao J., Yan J., Shang W. (2017). Berberine suppresses LPS-induced inflammation through modulating Sirt1/NF-κB signaling pathway in RAW264.7 cells. Int. Immunopharmacol..

[20] Kandikattu H.K., Rachitha P., Jayashree G.V., Krupashree K., Sukhith M., Majid A., Amruta N., Khanum F. (2017). Anti-inflammatory and anti-oxidant effects of Cardamom (Elettaria repens (Sonn.) Baill) and its phytochemical analysis by 4D GCXGC TOF-MS. Biomed. Pharmacother..

[21] Aziz R.S., Siddiqua A., Shahzad M., Shabbir A., Naseem N. (2019). Oxyresveratrol ameliorates ethanol-induced gastric ulcer via downregulation of IL-6, TNF-α, NF-ĸB, and COX-2 levels, and upregulation of TFF-2 levels. Biomed. Pharmacother..

[22] Shapouri-Moghaddam A., Mohammadian S., Vazini H., Taghadosi M., Esmaeili S., Mardani F., Seifi B., Mohammadi A., Afshari J.T., Sahebkar A. (2018). Macrophage plasticity, polarization, and function in health and disease. J. Cell. Physiol..

[23] Baatar D., Siddiqi M.Z., Im W.T., Ul Khaliq N., Hwang S.G. (2018). Anti-Inflammatory Effect of Ginsenoside Rh_2_-Mix on Lipopolysaccharide-Stimulated RAW 264.7 Murine Macrophage Cells. J. Med. Food..

[24] Jung Y.J., Lee J.E., Lee A.S., Kang K.P., Lee S., Park S.K., Lee S.Y., Han M.K., Kim D.H., Kim W. (2012). SIRT1 overexpression decreases cisplatin-induced acetylation of NF-κB p65 subunit and cytotoxicity in renal proximal tubule cells. Biochem. Biophys. Res. Commun..

[25] Chen D., Gong Y., Xu L., Zhou M., Li J., Song J. (2018). Bidirectional regulation of osteogenic differentiation by the FOXO subfamily of Forkhead transcription factors in mammalian MSCs. Cell. Proliferat..

[26] Giannakou M.E., Partridge L. (2004). The interaction between FOXO and SIRT1: Tipping the balance towards survival. Trends Cell. Biol..

[27] Cunningham M.A., Zhu Q., Hammond J.M. (2004). FoxO1a Can Alter Cell Cycle Progression by Regulating the Nuclear Localization of p27^kip^ in Granulosa Cells. Mol. Endocrinol..

[28] Chen P., Shi X., Xu X., Lin Y., Shao Z., Wu R., Huang L. (2018). Liraglutide ameliorates early renal injury by the activation of renal FoxO1 in a type 2 diabetic kidney disease rat model. Diabetes Res. Clin. Pract..

