# Supplementary material for: Sweroside Alleviated LPS-Induced Inflammation via SIRT1 Mediating NF-κB and FOXO1 Signaling Pathways in RAW264.7 Cells
Source: Molecules. 2019 Mar 1;24(5):872. doi: 10.3390/molecules24050872 (PMC6429084; doi:10.3390/molecules24050872)
Supplement: Supplementary file 1 [file molecules-24-00872-s001.zip › Supplementary Materials/Supplementary Materials.docx]

## Supplementary Materials

**Sweroside Alleviated LPS-Induced Inflammation via SIRT1 Mediating NF-κB and FOXO1 Signaling Pathways in RAW264.7 Cells**

Rui Wang ^1^, Zhaoyue Dong ^1^, Xiaozhong Lan ^2^, Zhihua Liao ^3^, and Min Chen ^1,*^

^1^ *College of Pharmaceutical Sciences, Key Laboratory of Luminescent and Real-Time Analytical Chemistry (Southwest University), Ministry of Education, Southwest University, Chongqing 400715, P.R. China;*

^2^ *TAAHC-SWU Medicinal Plant R&D Center, XiZang Agriculture and Animal Husbandry College，Nyingchi, Tibet 860000, P.R. China;*

*^3^ School of Life Sciences, Southwest University, Chongqing 400715, P.R. China*

^*^Correspondence: [mminchen@swu.edu.cn](mailto:mminchen@swu.edu.cn); Tel.: +86-23-68250579

**List of Contents**

**S1. Experiments**

1.1 Cytotoxic activity test of *P. hookeri* extract in RAW264.7 cells.

1.2 Anti-inflammatory test of *P. hookeri* extract in LPS-induced RAW264.7 cells.

**S2. Results**

2.1 Effects of cytotoxicity and anti-inflammatory activity on *P. hookeri* extract.

Fig. S2 Effects of cytotoxic activity and anti-inflammation on *P. hookeri* extract in RAW264.7 cells.

**S1. Experiment**

- 1. *Cytotoxic activity test of P. hookeri extract in RAW264.7 cells.*

About 5000 RAW264.7 cells per well were seeded into 96-well plates containing complete DMEM (100 μL) and continuously cultured for 12 h in incubator. Following the diverse concentration (20, 40, 80, 160 and 320 μg/mL) of *P. hookeri* extract for 24 h, every well was supplemented with 10 μL of CCK-8 solution and incubated at 37 °C for another 4 h. The normal control cells were set in Con group. The absorbance at 450 nm was assessed by a multifunctional microplate reader.

*1.2 Anti-inflammatory test of P. hookeri extract in LPS-induced RAW264.7 cells.*

RAW264.7 cells were seeded in 48 wells plate at a density of about 8000 per well with 200 μL complete DMEM for 12 h until adherence. The low, middle and high dosage of *P. hookeri* extract (40, 80 and 160 μg/mL) were added to LPS (1 μg/mL)-induced RAW264.7 cells for 24 h. The normal control cells were set in Con group. The level of NO in each group were determined by the NO assay kit with the multifunctional microplate reader. All the procedures were according to the manufacturer’s instructions.

**S2. Results**

*2.1* *Effects* of cytotoxicity and *anti-inflammatory activity on P. hookeri extract.*

The cytotoxicity assay indicated that *P. hookeri* extract exhibited no obvious cytotoxicity within the concentration 0-160 μg/mL. While there was a weak cytotoxicity (cell viability at 82.2%) at 320 μg/mL (Fig. S2A). In further anti-inflammation research, 40, 80 and 160 μg/mL were set as the low, middle and high dosage, respectively. Our results showed that the extract could significantly reversed the NO production in LPS-induced RAW264.7 cells for 24 h, especially at the dosage of 160 μg/mL (*P* < 0.01, Fig. S2B).


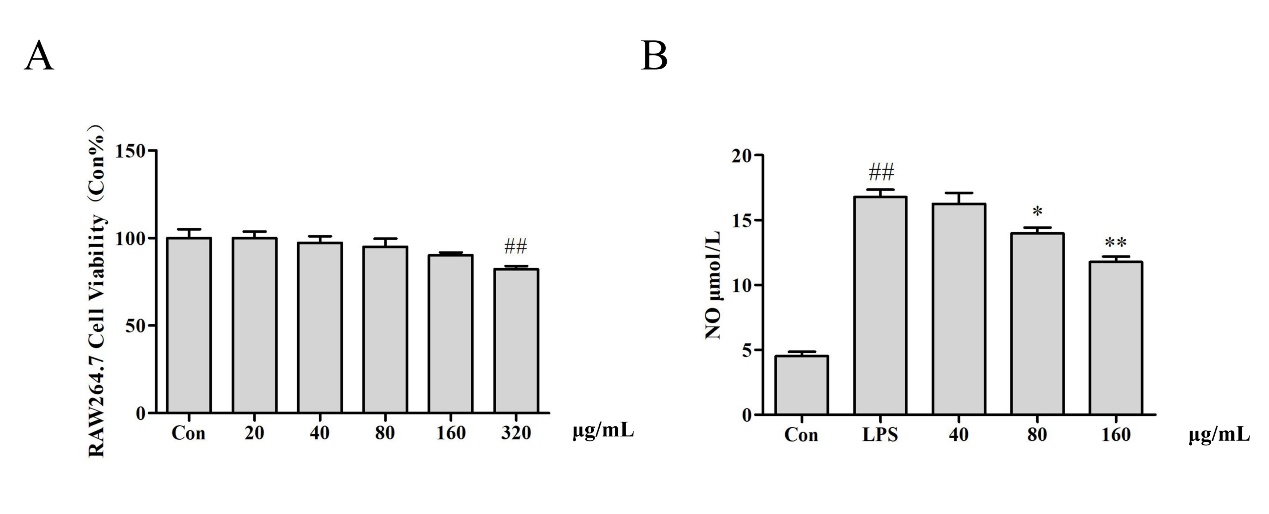


**Fig. S2 (A)** The cytotoxicity assay was conducted to measure the viability of RAW264.7 cells with *P. hookeri* extract (20-320 µg/mL) for 24 h. **(B)** The NO assay was detected with the treatment of *P. hookeri* extract (40, 80 and 160 μg/mL) in LPS-induced RAW264.7 cells for 24 h. Values expressed as mean ± SD (n = 5), ^#^*P* < 0.05, ^##^*P* < 0.01 compared with the Con group; ^*^*P* < 0.05, ^**^*P* < 0.01 compared with the LPS group.
