# Supplementary figures and images for: Sweroside Alleviated LPS-Induced Inflammation via SIRT1 Mediating NF-κB and FOXO1 Signaling Pathways in RAW264.7 Cells
Source: Molecules. 2019 Mar 1;24(5):872. doi: 10.3390/molecules24050872 (PMC6429084; doi:10.3390/molecules24050872)

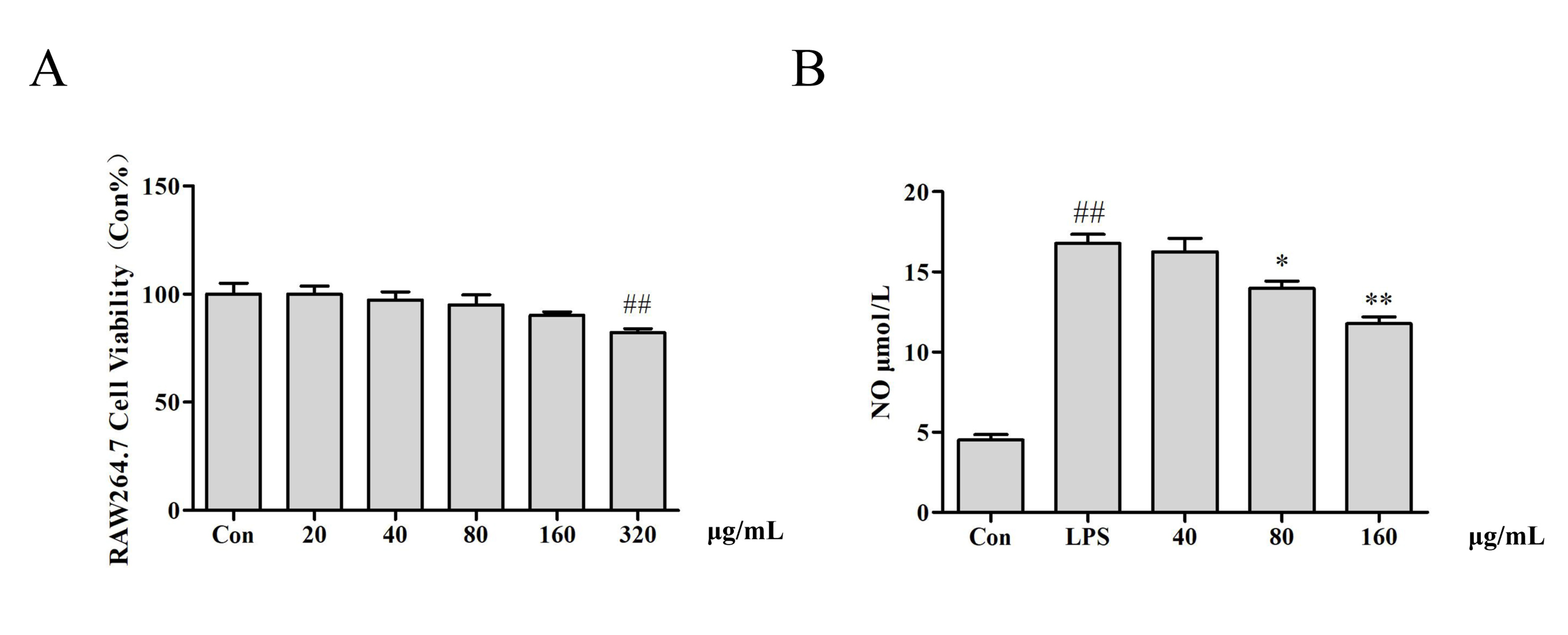

Supplement: Supplementary file 1 [file molecules-24-00872-s001.zip › Supplementary Materials/fig S2.jpg]
